# Hospital and Institutional News

**Published:** 1919-11-01

**Authors:** 


### HOSPITAL AND INSTITUTIONAL NEWS.

### G. Q. ROBERTS, C.B.E., M.A.OXON.

It will create universal sympathy to learn that Mr. Roberts has undergone a very severe operation, which in God's mercy he has borne without suffering any drawback, so that the patient has made wonderful progress from the day he underwent it. For some time, uncomplainingly and quietly, Mr. Roberts has suffered a great deal of pain and trouble, which were much aggravated by the excessive work of the last few years. When at length he consulted his physician, immediate measures were adopted, with the result that it was found necessary to operate without a moment's further delay. He is recovering from the shock of the operation, doing well in fact, and there is every reason to hope that the result of it will be to leave him stronger and better than he has been for many years. All his fellow workers, and all who are interested in the hospitals of this country, as well as those who have had business with St. Thomas's Hospital, will extend their warmest sympathy to Mr. Roberts in the circumstances, and will pray for his speedy recovery and quick return to complete health. The war has been a great searcher for trouble amongst all who have devoted their energies to the vast variety of work it has created for all loyal men and women throughout the Empire. Mr. Roberts has been decorated by the King for his services; he has won for himself a multitude of warm friends, and the recognition and appreciation of many who occupy a high position in the hospital field. Most of our readers, and especially nurses of all grades, will sympathise very deeply with Mrs. Roberts in the burden of anxiety and continuous nursing which have so unexpectedly fallen to her lot.

### "A COMFORT TO THOSE WHOM WE TREAT AND TEND."

It is an interesting fact that a service was held in the chapel of the Radcliffe Infirmary and County Hospital, Oxford, on St. Luke's Day, when a heart-stirring address to members of the medical and nursing professions was given by the Rev. W. Lock, D.D., warden of Keble College, Oxford. It proved most helpful to all who heard it, and cannot fail to bring strength to all who minister to the sick, who may have the privilege to read it for themselves. It is printed in full on page 78 *et seq.* of *The Nursing Mirror* for November 1, 1919, and we commend it to all our readers, men and women, who take a serious view of their responsibilities and life-work; and which of us does not? Dr. Lock properly makes the point that both doctors and nurses will gain additional strength by realising that all who minister to the sick, by thinking of their patients, not as bringing comfort to themselves but to the patients whom each treats and tends, will come to regard themselves properly as being the agents through whom Christ works in bringing rest and refreshment to the doctor and the nurse. Dr. Lock has much that is interesting to say on St. Luke's recognition that it was the Lord who gave St. Paul his power, and he gave a lifelong devotion to St. Paul as to a risen Lord, who is still able to inspire them. This was the result upon a physician of seeing the healing power of St. Paul and of Christ himself. May it be given to each reader of The Hospital to become more and more devoted to Christ's service, and to go more heartily and hopefully henceforward about their work.

### FIRST MEETING OF CONSULTATIVE COUNCIL.

The first meeting has been held of the Consultative Council appointed by the Minister of Health (Dr. Addison) to advise on questions relating to medical and allied services. Sir Bertrand Dawson is the Chairman and Colonel C. J. Bond, of Leicester, the Vice-Chairman of the Council. There was a full attendance of members, and Dr. Addison opened the proceedings as President of the Council. After welcoming the members of the Council, Dr. Addison said that he was sure that the experiment which he was making in bringing a body of this kind into close touch with the work and plans of the new Ministry, would be justified by results. Their main duty would be to advise him on questions of policy in the formative stage. They would thus be brought into the counsels of the Minister before decisions were taken, and if their advice was to have full weight, the confidential character of their relations with the Minister must be respected. At the same time, he had to remember that the Council must be put in a position to keep him in touch with the medical profession throughout the country, and to help him to carry the profession with him in the policy which he adopted. Therefore, he did not propose that the whole of their work should be conducted in secret. He had agreed with Sir Bertrand Dawson that periodical statements should be issued to the Press of the nature of the problems referred to the Council, and of the progress made with their deliberations, and he hoped that this arrangement would encourage discussion and criticism. He asked the Council at once to consider a reference which would enable them to tell him what in their view should be the ideal system of medical and allied services towards which the Ministry should work. Obviously this was a vast subject, and he would leave them completely free to consider its various aspects in order of urgency.

### A NEW INSTRUMENT OF GOVERNMENT.

Sir Bertrand Dawson, after thanking Dr. Addison for his cordial reception of the Council, said that in bodies of this kind he saw a new instrument of Government full of possibilities. The growth of knowledge and the necessity for securing public confidence in proposals which closely affected large sections of the population had made the development of some such organ of communication inevitable. This did not mean that Departmental advice was not good; but that it needed supplementing from new sources. The Consultative Councils were an experiment devised to meet this need. The problems referred to them would tax their powers to the full, and in feeling their way to the best methods of working they would welcome and rely upon that co-operation which they were promised by Dr. Addison and his advisers. In his opinion the activities of the Ministry of Health were bound to extend and to affect all branches of medicine more and more closely. If the results were to be satisfactory, a good understanding and close co-operation with the medical profession must be maintained throughout. He hoped that the profession would find in the Council a new link with the Minister. They would not displace any existing organisation in the counsels of the Ministry, but would endeavour to represent the views of all sections of the profession. He was deeply conscious of the honour conferred upon him by the Minister's invitation to act as Chairman of so distinguished a body. The responsibility was a heavy one; but with the help of his colleagues and the support of Dr. Addison and his advisers he did not doubt that they could take an effective share in the shaping of the new policy which the Minister of Health had been appointed to carry out. Dr. Addison then asked Sir Bertrand Dawson to take the chair, and after making arrangements as to their future procedure the Council held a preliminary discussion of the principles to be observed in the future developments of medical and allied services upon which the Minister had asked them to advise.

### THE ROYAL SANITARY INSTITUTE.

On Friday, November 14, at 7 p.m., in the Town Hall, Bootle, a discussion will take place on the Reconstruction of the Public Health Service. Dr. E. W. Hope, Medical Officer of Health for Liverpool, will open the discussion and the chair will be taken by Sir Henry Tanner, C.B., Chairman of the Council. On Saturday, November 15, visits will be arranged to the School Clinics, Maternity and Child Welfare Centre (including Remedial Exercises and Electrical Department), and also to certain industrial premises.

### A STRIKING APPEAL.

Many a householder in London would rub his eyes upon receiving a few days ago through the post what looked like a copy of one of the daily illustrated papers, but which, after a second look, was found to be No. 1 of *Bart's Illustrated Chronicle*. In this extraordinarily interesting appeal or periodical there are sixteen pages of pictures and reading matter, the pictures wisely predominating, all about St. Bartholomew's and its gigantic appeal. The whole get-up of the effort is most striking and the simulation of the headlines and the clever paragraphing of the popular press makes the illusion most complete. We notice that the appeal has been compiled by "Business Builders, Limited," 26 Great Ormond Street, from which thoroughfare, curiously enough, was issued a few days ago another of the colossal appeals now before the public. The idea of the periodical appeal on the part of a hospital is not new; it was done on the occasion of the jubilee of the National Hospital for the Paralysed and Epileptic in 1909, but not on such a large scale. Appeal advertisement on the part of our charities is becoming a high art, one of the best examples of which is the gigantic hand writing out the mammoth cheque on the hoarding outside the Middlesex Hospital.

### MEDICAL SCHOLARSHIPS FOR BOYS AND GIRLS.

In the will of Mr. Francis Fletcher, of Sutton Coldfield, a director of Wyley's, Limited, manufacturing chemists, provision is made for scholarships for boys and girls desirous of entering the medical profession. The scholarships, which are of the value of £100, and tenable for five years, are to be called the "Frank Fletcher Scholarship for Boys" and the "Catherine Fletcher Scholarship for Girls." The scholars are to be the children of parents belonging to the industrial classes who are earning a daily or weekly wage not exceeding 50s. a week. They are to be selected from those attending public elementary schools, or for boys from grammar schools and girls from municipal high schools who have won their way from the lower-grade schools, but they must be sons and daughters of *bona-fide* working men and women. The testator expresses the opinion in his will that "there are potentialities of the born doctor being found amongst such children," in which case it would be in the interest of medical and surgical science, also to the benefit of humanity, believing as I do that the service of man is the highest worship of God." Mr. Fletcher's provision is very generous, but the high cost of living would make it difficult for the scholars to pursue their medical studies unless the medical schools happened to be in the same city as the family home, or unless the scholarship could be augmented from other sources.

### THE TRAVELLING HOSPITALS OF EGYPT.

The annual report of the director of ophthalmic hospitals in Egypt to the Department of Public Health expresses the opinion that, owing to the large number of blind in Egypt, it would be a highly uneconomical thing to undertake charitable provision for them on any large scale while the prevention of blindness is so meagrely endowed; the prevention of blindness should be by provision of hospitals where early cases of acute conjunctivitis can be dealt with. There are thirteen permanent hospitals in Egypt and these are aided by four travelling hospitals, two of which were endowed by Sir Ernest Cassel. The numbers of patients treated are remarkable, 81,529 new patients were treated in 1917, an increase of 19 per cent. on 1916, the attendances of out-patients were 1,004,161, and 59,581 operations were performed. Thirteen per cent. of all patients examined were blind in one or both eyes, mostly through acute conjunctivitis or ophthalmia. That the travelling hospital system is considered sound in some provinces is shown by the fact that projects for further provision are being discussed.

### WAS NAPOLEON RESPONSIBLE?

It has generally been supposed that the epidemic of ophthalmia which affected so disastrously the French troops in Napoleon's Egyptian campaign of

1799 to 1801 was a trachomatous infection, and this point, side by side with the cause of ophthalmia among British troops in the Egypt Expeditionary Force 1916-1918, was examined by Lt.-Col. H. L. Eason in a paper read before the Ophthalmological Society of Egypt. Early last century people were of opinion that trachoma amongst European armies had been introduced into Europe from Egypt (hence *Ophthalmia egyptiaca*) by Napoleon, but subsequent historical researches showed that the disease had already been epidemic in Europe since antiquity. But when, by reason of the Napoleonic wars, the armies came so repeatedly in contact with each other and with the civil population, it became frightfully prevalent. In the English army, during the year 1818, there were more than 5,000 on the invalid list who had been rendered blind as a consequence of trachoma, and in contemporary armies things were as bad or worse. Larrey's "Mèmoires de Chirurgie Militaire et Campagnes" clearly attributes the eye troubles of Napoleon's soldiers in Egypt to climatic conditions. He made the curious observation that of those affected by ophthalmia nearly all who lost the sight of one eye lost the sight of the left, and attributes this in part to the fact that as most people sleep on the right side, that part is more affected by the humidity of the earth. In the recent British expedition there was little trachoma. Col. Eason had details of 279 cases, of which only 63 were recent, the rest having been acquired before the soldiers arrived in Egypt. Ophthalmia generally caused about 10 per cent. of the total eye cases; he draws the conclusion from his researches that ophthalmia of any sort is not a serious danger to an army with modern standards of sanitation, and this is strengthened by its incidence upon the opposing Turkish forces.

### THE FOOD CONTROLLER ONCE MORE.

Our old friend the Register of Consumption has made its appearance again, and institutions which have not been exempted by the Food Controller from keeping a register must presumably obtain exemption or obtain a register and set it going. The book is not quite so troublesome as it used to be, as only three articles are included, *viz.* Government butter, sugar, and butchers' meat. As nobody nowadays, except millionaires and war profiteers, can afford to eat butter the register in the case of institutions is further simplified. It is our steady policy on all occasions to uphold the law, but we are curious to know if, during the former keeping of the registers, they were ever examined by anyone in authority and if they were found to answer any useful purpose. We are told by a secretary of a large hospital that during the war it was his custom carefully to lose all forms sent in by the various controllers, and it was a curious fact that in nine cases out of ten he was never troubled in the matter again.

### HOSPITAL CLAIM FOR COMPENSATION.

At a recent sitting of the War Losses Commission a battle of wits took place between Mr. Charles Cutting, Secretary of the Royal Hospital for Incurables, Putney, and Mr. R. Hopcroft, of the Office of Works. It will be remembered that the Royal Hospital's City office in St. Paul's Churchyard had to be evacuated in a hurry to make room for one of the numerous Government Departments. A new abiding place was found in Bond Court House, Walbrook, and the Royal Hospital claims a sum of about £2,000 in respect of rent, cost of removal, etc. Mr. Cutting was asked why an office in the City was necessary, and he replied that in the instance of his charity it is a front trench, a good place to appeal from. Many calls were received from subscribers in the City, Stock Exchange men and others, who would not bother to go to Putney. The Office of Works representative had offered premises in exchange in Queen Victoria Street, but Mr. Cutting had retorted that "Queen Victoria Street compared with Ludgate Hill is like Brick Lane to Piccadilly." The hospital did not wish to return to St. Paul's Churchyard, as a further change of address would lead to confusion in the minds of the supporters. It was not explained why the Government could not themselves take the premises in Queen Victoria Street.

### STATISTICS OF GERMAN PEOPLE'S LOSSES.

The Danish Society for investigation of the social effects of the war has issued a series of brochures showing statistics of the ravages sustained by the population of the countries engaged. Take Germany to begin with:—"The German people suffered through the decrease of births and increase of mortality a total loss of 5,600,000 human beings. The number of inhabitants (in round figures) has sunk from 67,800,000 to 65,100,000, of whom 33,900,000 are females, and only 31,200,000 of the male sex. Of the total loss of 5,600,000, 3½ millions are due to the falling of births and 2,100,000 to the increase of mortality." No fewer than 1,800,000 Germans have lost their lives in the war, while thousands of the survivors have been more or less severely crippled. And through this "inverted race-selection the best labour power of the German people has for the greater part been destroyed." Through under-nourishment and overwork the health condition of the population has been enormously worsened, and as a consequence of economic difficulties one may anticipate an abnormal falling-off in the birth-rate and an abnormal rate of mortality. Are there no statistics available of the nation's power of recovery? France after 1870 belied the pessimistic prophets.

### SAFETY FIRST MOVEMENT IN THE FACTORY.

In the annual report of the Chief Inspector of Factories for 1918 we find that only one-third of the accidents for that year were due to machinery. Of these less than 35 per cent. were due to the absence of a guard. Negligence and want of thought, lack of appreciation of the danger, are responsible for the two-thirds. It is felt strongly that the condition of things will not be remedied by Act of Parliament alone. In this as in other matters we must have the co-operation of both employers and employees in their determination to bring about a reduction of accidents. The Safety First movement should have good results both in the street and in the factory and workshop. Many firms are forming their own Safety Committees to deal with this problem. It has been found that meetings stimulate interest, and the workpeople have already volunteered many useful suggestions as to safety devices and their proper use.

### THE CORONER'S COURT.

Dr. Waldo is a coroner of great experience and one whose views are entitled to every respect. Last month several of his expressed views on the subject of inquests are well worthy of mention. He said that, as was his custom, he had merely read in Court two or three material, relevant lines from the bulky correspondence found on the body of the deceased. The jury and others interested in the case had had an opportunity of perusing the documents in full. The reading of details in Court lead to their publication by the Press, which not only gave pain and distress to the relatives, but often led to further suicides by suggestion and incitation. For example, a short time ago, three brothers, one after another, took their own lives by placing their heads in the same stove with the gas turned on. A lessening in the number of suicides would undoubtedly follow the suppression by the Press of detailed reports of sensational and "interesting" cases of suicide. *If* any class of case might advantageously be held in private by coroner and jury, to the exclusion of the Press and other members of the public, probably it is that of a certain number of selected cases of suicide. It was not, however, for a moment suggested that cases in which the good name of an individual was at stake should be held other than in the presence of Press and public. Also the return of weapons, such as pistols, knives, ropes, etc., by which suicide was accomplished, to relatives might, in some cases, act injuriously by suggestion and incitation. Generally he stands as a great believer in the usefulness of full publicity of the Coroner's Court, and, with him, we trust that before long the pre-war constitutional and uniform method of sitting in all cases of inquisition with a jury will be resumed.

### DISCHARGES FROM SANATORIA.

At a meeting of the London Insurance Committee at Spring Gardens, Mr. Bowden moved that agreements entered into with various sanatoria should provide for varied food, also that persons entering such institutions should be advised to remain there as long as the medical officer deemed desirable. An enormous number of sanatoria patients took their discharge voluntarily before the completion of their treatment. At one hospital, from which there had been several complaints, it was said the patients were served with roast mutton for thirteen weeks without a change. Mr. Marshall also complained that the food was monotonous. It was not lacking in quantity or quality, but was badly prepared. Members of the Committee agreed inquiry ought to be made as to the reasons for early discharges. Mr. Handel Booth said the women did not discharge themselves. It was the men. Their reasons for doing so were connected mostly with beer and tobacco. They might investigate for years, and they would come to that at the finish. Probably if they fed these men on pork-pies and pickles they would be satisfied (laughter), but even in country houses with kitchen gardens they complained because they were away from picture houses and public houses.

### WHAT CARRIED THE DAY.

The St. Austell Guardians lately sought to fill the post of a part-time temporary medical officer of health for the workhouse and the rural district council, for which applicants were invited to name the salary which they required. Six medical men applied, and severally named £800, £550, £500, £250 (two), and £190. Those naming the two lowest sums were three local practitioners, of whom the only one who had been on active service was appointed. The matter is interesting for several reasons. Dr. E. G. Andrews, because he had been on active service, carried the day. The gentleman who offered to accept the lowest salary was not appointed. The salaries demanded varied much. Yet, with the fall in the purchasing power of money, a salary of £250 for a medical post, held previously by a whole-time officer, is moderate. The appointment must be justified by results, but the St. Austell Guardians can claim the honour of not having made their choice upon commercial or narrow-minded grounds. It is always pleasant to find the hospital standard in the Poor Law; and to find it there is not any longer (thanks in part to Dickens) a surprising exception to the rule.

### GREY'S HOSPITAL, PIETERMARITZBURG.

The new wing being built at Grey's Hospital, Pietermaritzburg, Natal, is approaching completion. The building is provided with a lift, is estimated to hold sixty patients, and it imparts to the institution the character of a first-class hospital as such hospitals are counted in the Union. The building is of two storeys, cool and cheerful, and has a tiled roof. New Mental Hospital buildings are being erected at Queenstown, Cape Province.

### THIS WEEK'S DRUG MARKET.

A fair business continues and on the whole prices are maintained with an advancing tendency in several cases. The paralysing strikes in the United States cannot fail to affect the prices here of American drugs unless the strikes are soon over. Potassium bromide of German make is now being offered on this market, but at present supplies are insufficient and the price is maintained. Calomel, corrosive sublimate, and other salts of mercury are lower; this was not unexpected; the quicksilver market is still unsettled, and therefore the prices of mercurials cannot yet be regarded as stable. English refiners of camphor have again raised their prices. Opium has an upward tendency in value, and clove oil is again dearer. Eucalyptus oil is in good demand and prices are firmly maintained. Castor oil, honey, phenazone, and valerian are among the few articles with a downward price tendency.

